# Reliability, ease of use and usefulness of I-MeDeSA for evaluating drug-drug interaction alerts in an Australian context

**DOI:** 10.1186/s12911-018-0666-y

**Published:** 2018-10-05

**Authors:** Melissa T Baysari, David Lowenstein, Wu Yi Zheng, Richard O Day

**Affiliations:** 1 Centre for Health Systems & Safety Research, Australian Institute of Health Innovation, Macquarie University, Sydney Australia and St Vincent’s Clinical School, Faculty of Medicine, UNSW, Sydney, Australia; 2 School of Medical Science, Faculty of Medicine, UNSW, Sydney, Australia; 3 Centre for Health Systems & Safety Research, Australian Institute of Health Innovation, Macquarie University, Sydney, Australia; 4 Department of Clinical Pharmacology & Toxicology, St Vincent’s Hospital, Sydney Australia and St Vincent’s Clinical School, Faculty of Medicine, UNSW, Sydney, Australia

**Keywords:** Human factors compliance, Computerized alerts, Drug-drug interactions

## Abstract

**Background:**

Recently, attention has shifted to improving the design of computerized alerts via the incorporation of human factors design principles. The Instrument for Evaluating Human Factors Principles in Medication-Related Decision Support Alerts (I-MeDeSA) is a tool developed in the United States to guide improvements to alert design and facilitate selection of electronic systems with superior design. In this study, we aimed to determine the reliability, ease of use and usefulness of I-MeDeSA for assessing drug-drug interaction (DDI) alerts in an Australian context.

**Methods:**

Using the I-MeDeSA, three reviewers independently evaluated DDI alert interfaces of seven electronic systems used in Australia. Inter-rater reliability was assessed and reviewers met to discuss difficulties in using I-MeDeSA and the tool’s usefulness.

**Results:**

Inter-rater reliability was high (Krippendorff’s alpha = 0.76), however, ambiguous wording and the inclusion of conditional items impacted ease of use. A number of items were not relevant to Australian implementations and as a result, most systems achieved an I-MeDeSA score of less than 50%.

**Conclusions:**

The I-MeDeSA proved to be reliable, but item wording and structure made application difficult. Future studies should investigate potential modifications to the I-MeDeSA to improve ease of use and increase applicability to a variety of system configurations.

**Electronic supplementary material:**

The online version of this article (10.1186/s12911-018-0666-y) contains supplementary material, which is available to authorized users.

## Background

Drug-drug interactions (DDIs) occur when two or more drugs are taken concurrently and the result is a change in the effect of one or more of the drugs. DDIs can result in adverse effects (e.g. bleeding) or to one or both of the drugs not achieving their therapeutic effect [1]. DDIs are a significant cause of patient morbidity and mortality worldwide [2–5].

Despite being predictable in nature, potential DDI errors are often missed by prescribers and pharmacists [6]. The sheer volume of known drug interactions is likely to contribute to poor DDI detection. Electronic systems are increasingly being adopted by hospitals all over the world as a means of reducing medication errors, including DDIs. One of the core benefits of these systems is the ability to provide clinicians with information and guidance at the point of care. Computerised decision support can take many forms, the most common being computerised alerts. DDI alerts are often included as a form of decision support in electronic prescribing and dispensing systems to warn prescribers and pharmacists of potential DDIs [7]. Although frequently implemented, previous studies have shown that users override most DDI alerts presented [8–11]. That is, most alerts are clicked past without alert recommendations being followed.

A variety of factors are likely to be contributing to poor DDI alert acceptance, however alert design has been identified as one of the most important factors [10]. Alert design relates to multiple aspects of alert implementation, including the mechanisms underlying alert generation, alert visual appearance, and the options available to users to accept or reject alert recommendations. Poor alert design is a frequent complaint by users and is viewed as a priority area to enhance alert potential to improve medication safety [12–15].

Recently, attention has shifted to improving DDI alert design via the incorporation of human factors (HF) design principles [16]. In a recent series of studies, [16, 17] researchers in the United States developed a standardised tool for evaluating DDI alerts in terms of their compliance to HF principles [18]. This tool, the Instrument for Evaluating Human Factors Principles in Medication-Related Decision Support Alerts (I-MeDeSA; see Additional file 1), was developed to guide improvements to DDI alert design and facilitate selection of electronic systems with superior HF design [18].

The I-MeDeSA assesses compliance with nine HF design principles (see Table 1) and is composed of 26 items with binary scoring (i.e. 1 or 0 to indicate a yes or no answer). Initial validation of the tool involved content validation by three HF experts, pilot testing with three electronic medical record (EMR) systems, inter-rater reliability testing, and an evaluation of construct validity via the assessment of alerts in two EMRs of various ages [18]. A subsequent US study utilized the I-MeDeSA to evaluate 14 systems and showed that HF compliance was generally poor [19]. In one of the few applications of the I-MeDeSA outside the US, a Korean-language version of the tool was used by two medical informatics reviewers to assess DDI alerts in a Korean EMR [20]. The tool was found to be useful and generalizable but reviewers identified a number of problems with the tool, including the need for more concrete definitions, clearer rationale for each item and more explicit examples [20].

**Table 1:** Human factors principles assessed by the Instrument for Evaluating Human Factors Principles in Medication-Related Decision Support Alerts (I-MeDeSA) [18]

Human factors principle	Number of items in the I-MeDeSA that assess this principle
Alarm philosophy	1
Placement	4
Visibility	3
Prioritization	5
Colour	2
Learnability and confusability	1
Text-based information	6
Proximity of task components being displayed	1
Corrective actions	3

In this study, we aimed to assess the reliability, ease of use and usefulness of the I-MeDeSA for evaluating DDI alerts in an Australian context.

## Methods

### Electronic systems evaluated

Three reviewers, two HF researchers (MB, WYZ) and a medical science honours student (DL), utilised I-MeDeSA to assess DDI alert interfaces in seven electronic systems currently in use in Australian hospitals, primary care settings and pharmacies. Hospital computerised provider order entry (CPOE) systems were Cerner’s *PowerChart* (https://www.cerner.com/solutions/Hospitals_and_Health_Systems/Acute_Care_EMR/PowerChart/?LangType=3081), DXR Technology’s *MedChart* (http://www.dxc.technology/providers/offerings/139499/140202-medchart_electronic_medication_management), and InterSystem’s *Trakcare* (http://www.intersystems.com/our-products/trakcare/trakcare-overview-2/). Primary care EMR systems were *Best Practice* (http://www.bpsoftware.net/) and *Medical Director* (http://medicaldirector.com/), and pharmacy dispensing systems were *FRED* (https://www.fred.com.au/what-we-do/dispensary/fred-dispense/) and *iPharmacy* (http://www.rxone.com.au/dispense.html).

### Procedure

Prior to commencing formal data collection, the three reviewers undertook a pilot test of the I-MeDeSA. The three reviewers independently rated DDI alerts in the oncology information system *MOSAIQ* (Elekta) (https://www.elekta.com/software-solutions/care-management/mosaiq-radiation-oncology/), and then came together to discuss any issues or difficulties in utilizing the tool. This led to the identification of ambiguous terms in a number of items and a consensus was reached among reviewers on how these criteria would be applied during assessments.

For formal data collection, multiple site visits were undertaken to hospitals, clinics and offices to evaluate the DDI alerts in each system. Reviewers received a ‘walk through’ of each system by an experienced user or administrator who also answered any queries about alerts that could not be ascertained from a demonstration of the system (e.g. whether a catalogue of unsafe events was available to users). To generate DDI alerts in each system, reviewers provided demonstrators with a list of drug pairs known to potentially interact. Both major and minor DDIs were inputted into systems during demonstrations. Reviewers took hand-written notes during walk-throughs and were provided with screenshots of DDI alerts to assist with subsequent evaluations.

Following each demonstration, reviewers independently assessed DDI alerts in each system using I-MeDeSA. Reviewers then came together to discuss scores, to reach a consensus on a final score for each system if disagreements arose, and to identify any additional difficulties or problems encountered while using the I-MeDeSA.

### Inter-rater reliability

To determine inter-rater reliability between the three reviewers, Krippendorff’s alpha was used to assess consistency between reviewers on overall I-MeDeSA scores awarded to the seven alert interfaces.

## Results

### I-MeDeSA reliability

Reviewers were highly consistent in their application of I-MeDeSA. Krippendorff’s alpha found to be 0.7584, 95% CI [0.7035, 0.8133].

### I-MeDeSA ease of use

Reviewers identified two primary issues with the I-MeDeSA tool. Firstly, phrasing of a number of items was perceived to be ambiguous, leading to differences in how the items were interpreted by reviewers. Items identified to be problematic appear in Table 2.

**Table 2:** I-MeDeSA [18] items perceived to be ambiguous leading to differences in interpretation among reviewers

I-MeDeSA item	
(2i) Are different types of alerts **meaningfully grouped** (e.g., by the severity of the alert, where all Level 1 alerts are placed together, or by medication order, where alerts related to a specific medication order are grouped together)?	
(3iii) Is the font used to display the textual message **appropriate** for the user to read the alert easily (e.g., a mixture of upper and lower-case lettering is easier to read than upper case only)?	
(4iii) Are signal words **appropriately** assigned to each existing level of alert (eg, ‘Warning’ would appropriately be assigned to a Level 1 alert and not a Level 3 alert)? ‘Note’ would appropriately be assigned to a Level 3 alerts, and not a Level 1 alert.	
(5ii) Is color **minimally** used to focus the attention of the user? Excessive coloring used on the screen can create noise and distract the user. Therefore, colors should be kept to fewer than 10.	
(6i) Are the different severities of alerts **easily distinguishable** from one another? For example, do major alerts possess visual characteristics that are distinctly different from minor alerts? The use of a signal word to identify the severity of an alert is not considered to be a visual characteristic.	

Bolded words indicate components of items that were perceived to be ambiguous

Secondly, several of the I-MeDeSA items included related items so that a negative score on one item automatically impacted the score on another. That is, some items were automatically scored zero if a preceding item was not scored a one. Some examples appear in Table 3. As a result of the inclusion of conditional items, some systems were penalized multiple times for missing a single design feature. In total, 5 (20%) items were ‘related’ to preceding items.

**Table 3:** Examples of conditional items in the I-MeDeSA [18]

**(4i) Is the prioritization of alerts indicated appropriately by color (e.g. colors such as red and orange imply a high priority when compared to colors such as green, blue and white)?** (4ii) Does the alert use prioritization with colors other than green and red, to take into consideration users who may be colorblind?	
**(7iii) Does the alert include an instruction statement telling the user how to avoid the danger or the desired action?** (7iiia) If yes, does the order of recommended tasks reflect the order of required actions?	

Bold text indicates major item to which one or more other items are dependent

### I-MeDeSA usefulness

Evaluation of the seven DDI alert interfaces revealed that scores were low, with the majority (five of seven interfaces) scoring 50% or less. The average I-MeDeSA score was 49%. The I-MeDeSA proved to be useful in identifying several areas where alerts were non-compliant with HF principles, including for example, placement and corrective actions (see Additional file 2). However, the items in I-MeDeSA relating to prioritization were not relevant to all systems evaluated as some had only one level of DDI alert in place. In the same way, items relating to other alert types (e.g. allergy alerts) were not relevant for systems that only had DDI alerts operational. In total, 8 (31%) items were applicable to only some systems, contingent on the configuration in place.

## Discussion

Evaluation of seven systems using the I-MeDeSA allowed reviewers to identify a number of design issues that may be contributing to poor alert acceptance in Australian settings. Most systems achieved I-MeDeSA scores of less than 50%. However, due to the tool’s structure and content, systems were penalised multiple times for missing a single design feature and approximately a third of the items were not relevant to the system configurations in use in Australia.

Reviewers perceived a key difficulty with I-MeDeSA to be the use of ambiguous wording in some items, which led to differences in interpretation and inconsistent scores. For example, what one reviewer perceived to be an ‘appropriate’ font, another reviewer considered to be inappropriate. The inclusion of ambiguous items was also identified to be a problem in a previous study where I-MeDeSA was utilized to assess Korean DDI alerts [20]. As was the case in previous studies utilising I-MeDeSA, [18–20] inter-rater reliability was high in our application, however this was likely to be due to the in-depth discussions held during piloting. For example, during piloting, item 2i) ‘*Are different types of alerts meaningfully grouped?*’ was found to be problematic as reviewers interpreted ‘meaningful’ differently. Disagreements arose when one reviewer judged alphabetical grouping of interactions to be meaningful, while another disagreed, focusing instead on grouping interactions by severity level. Discussion between reviewers was required to come to an agreement on what the term ‘meaningful’ encompassed for subsequent assessments. If I-MeDeSA is intended to be used as an ‘off-the-shelf’ instrument by a single reviewer in the absence of rigorous pilot testing, more explicit terms and examples are needed to minimize confusion and facilitate more consistent application of the tool. For example, the instrument could specify the conditions under which a font is considered ‘appropriate’ for an alert (e.g. size, colour, style, etc).

Another factor that impacted ease of use of the I-MeDeSA was the use of conditional scoring (i.e. scoring a one (‘yes’) for a number of I-MeDeSA items was dependent on scoring a one for a preceding item). This scoring system penalized systems multiple times for missing a single design feature (e.g. the absence of colour) and in turn contributed to poor HF compliance scores. Similar concerns were raised by Cho et al. [20]. To ensure I-MeDeSA scores reflect true HF compliance, denominators should be revised based on the applicability of dependent items. For example, if a conditional item is not applicable to a system because the parent item has been marked ‘no’, the system should be scored out of 25 for HF compliance, not 26.

The main factor perceived to have impacted on the usefulness of the I-MeDeSA was the inclusion of irrelevant items. A key problem was identified to be that some items assumed that systems included alert types of various levels of severity. Five items in the tool relate to prioritization of alerts, and assess whether more severe alerts are easily distinguishable from less severe alerts (i.e. with colours, shapes and words). A number of systems evaluated in this study had only one level of DDI alert in place. Although technically possible to include multiple levels, the systems were tested in situ and sites had chosen to implement only ‘severe’ DDI alerts, so as to minimise the risk of user frustration and alert fatigue [21]. Adopting larger numbers of alerts with clear prioritization is not more complaint with HF principles than adopting fewer, more meaningful alerts. Thus, it seems counterintuitive to penalise these systems for not prioritizing alerts. In the Korean application of the I-MeDeSA, it also proved difficult to assess systems that did not employ multiple severity levels, and the authors suggested that a branch question be included at the start of the tool [20]. Assessing systems on only one level of alert severity would allow comparisons to be made across all systems, regardless of configuration. However, results from these comparisons would provide limited information on whether systems with multiple levels of alert severity use techniques to assist users to distinguish between these levels.

Similarly, several items in the I-MeDeSA relate to other alerts operational in systems (e.g. allergy alerts) and assess whether DDI alerts are easily distinguishable from other alert types. These items are not relevant in implementations that include only DDI alerts and so an additional branching question is also needed for this. Filter questions are important elements of good survey/tool design as they guide respondents away from questions that are not applicable [22]. Ideally, multiple branches, via the inclusion of appropriate filter questions, should be made available in the I-MeDeSA, with alert configuration dictating which branch is followed. This would make alert assessment more logical and streamlined, but would make comparisons of I-MeDeSA scores across systems with variable alert configurations more difficult.

## Conclusions

Overall, our results indicate that computerised alerts in use in Australian healthcare settings require significant redesign to incorporate human factors principles of good warning design. As a tool for assessing computerised medication alerts, the I-MeDeSA is reliable but suffers from several problems that negatively impact on ease of use and usefulness. Although a need clearly exists for a tool that allows easy assessment of HF compliance of computerised alerts, additional work is needed to ensure this US instrument is useful for evaluating alerting systems currently being used in other healthcare contexts, like Australia. In moving forward, we plan to adopt an evidence-based approach to guide the development of a more user-friendly and useful tool for alert evaluation.

## Additional files

Additional file 1: I-MeDeSA. This is a table including all items of the I-MeDeSA [18] and their descriptions. (DOCX 18 kb)

Additional file 2: I-MeDeSA scores for all systems in terms of human factors principles assessed. This is a table which includes a breakdown of the scores obtained by the seven electronic systems we assessed. (DOCX 94 kb)

## Abbreviations

CPOE: Computerised Provider Order Entry
DDI: Drug-Drug interaction
EMR: Electronic Medical Record
HF: Human Factors
I-MeDeSA: Instrument for Evaluating Human Factors Principles in Medication-Related Decision Support Alerts
