# Supplementary material for: Reliability, ease of use and usefulness of I-MeDeSA for evaluating drug-drug interaction alerts in an Australian context
Source: BMC Med Inform Decis Mak. 2018 Oct 5;18:83. doi: 10.1186/s12911-018-0666-y (PMC6173853; doi:10.1186/s12911-018-0666-y)
Supplement: Supplementary file 2 — I-MeDeSA scores for all systems in terms of human factors principles assessed. This is a table which includes a breakdown of the scores obtained by the seven electronic systems we assessed. (DOCX 94 kb) [file 12911_2018_666_MOESM2_ESM.docx]

**Additional files**

Additional file 1. I-MeDeSA (Zachariah *et al.*, 2011)

| **Human factors principle** | **Item numbers and descriptions** |
| --- | --- |
| Alarm Philosophy | **1i)** Does the system provide a general catalogue of unsafe events, correlating the priority level of the alert with the severity of the consequences? |
| Placement | **2i)** Are different types of alerts meaningfully grouped? |
|  | **2ii)** If available, is the response to the alert, indicating the user’s intended action (e.g. *Accept, Cancel/Override*), provided along with the alert, as opposed to being located in a different window or in a different area on the screen? |
|  | **2iii)** Is the alert linked with the medication order by an appropriate timing? |
|  | **2iv)** Does the layout of critical information contained within the alert facilitate quick uptake by the user? Critical information should be placed on the first line of the alert or closest to the left side of the alert box. Critical information should be labelled appropriately and must consist of: (1) the interacting drugs, (2) the risk to the patient, and (3) the recommended action. |
| **Visibility** | **3i)** Is the area where the alert is located distinguishable from the rest of the screen? |
|  | **3ii)** Is the background contrast sufficient to allow the user to easily read the alert message? |
|  | **3iii)** Is the font used to display the textual message appropriate for the user to read the alert easily? |
| Prioritisation | **4i)** Is the prioritisation of alerts indicated appropriately by colour? |
|  | **4ii)** Does the alert use prioritisation with colours other than green and red, to take into consideration users who may be colour blind? |
|  | **4iii)** Are signal words appropriately assigned to each existing level of alert? |
|  | **4iv)** Does the alert utilise shapes or icons in order to indicate the priority of the alert? |
|  | **4v)** In the case of multiple alerts, are the alerts placed on the screen in the order of their importance? |
| Colour | **5i)** Does the alert utilize colour-coding to indicate the type of unsafe event? |
|  | **5ii)** Is colour minimally used to focus the attention of the user? |
| Learnability & confusability | **6i)** Are the different severities of alerts easily distinguishable from one another? |
| Text-based information | **7i)** Does the alert possess a signal word to indicate the priority of the alert (e.g. ‘note’, ‘warning’, or ‘danger’?) |
|  | **7ii)** Does the alert possess a statement of the nature of the hazard describing why the alert is shown? |
|  | **7iia)** If yes, are the specific interacting drugs explicitly indicated? |
|  | **7iii)** Does the alert possess an instruction statement telling the user how to avoid the danger or the desired action? |
|  | **7iiia)** If yes, does the order of recommended tasks reflect the order of required actions? |
|  | **7iv)** Does the alert possess a consequence statement telling the user what might happen if the instruction information is ignored? |
| Proximity of task components | **8i)** Are the informational components needed for decision-making on the alert present either within or in close spatial and temporal proximity to the alert? |
| Corrective actions | **9i)** Does the system possess corrective actions that serve as an acknowledgment of having seen the alert while simultaneously capturing the user’s intended action? |
|  | **9ia)** If yes, does the alert utilise intelligent corrective actions that allow the user to complete a task? |
|  | **9ii)** Is the system able to monitor and alert the user to follow through with corrective actions? |

Additional file 2. I-MeDeSA scores for all systems in terms of human factors principles assessed

| **I-MeDeSA Principle** | **Interface score** | | | | | | | **Average score (%)** |
| --- | --- | --- | --- | --- | --- | --- | --- | --- |
|  | **1** | **2** | **3** | **4** | **5** | **6** | **7** |  |
| Alarm Philosophy (/1) | 0 | 0 | 0 | 0 | 0 | 0 | 0 | 0 (0) |
| Placement (/4) | 2 | 3 | 3 | 2 | 3 | 4 | 3 | 2.9 (73) |
| Visibility (/3) | 3 | 3 | 3 | 3 | 3 | 3 | 3 | 3.0 (100) |
| Prioritization (/5) | 0 | 3 | 1 | 0 | 0 | 0 | 0 | 0.6 (12) |
| Colour (/2) | 1 | 1 | 1 | 1 | 1 | 1 | 2 | 1.1 (55) |
| Learnability and Confusability (/1) | 0 | 1 | 0 | 0 | 0 | 0 | 0 | 0.1 (10) |
| Text-based information (/6) | 2 | 5 | 0 | 5 | 5 | 5 | 3 | 3.6 (60) |
| Proximity of task components being displayed (/1) | 1 | 1 | 1 | 0 | 0 | 1 | 1 | 0.7 (70) |
| Corrective actions (/3) | 1 | 1 | 1 | 1 | 0 | 1 | 1 | 0.9 (30) |
| **Total (/26)** | **10** | **18** | **10** | **12** | **12** | **15** | **13** | **12.9 (49)** |
